# Symptoms experienced during the first 4 months of chemotherapy administration: a longitudinal cohort study in an Australian cancer treatment unit

**DOI:** 10.1007/s00520-026-10955-w

**Published:** 2026-07-03

**Authors:** Kimberly E. Alexander, Theodora Ogle, Brighid Scanlon, Morgan J. Farley, Natalie Bradford, Erin Pitt, Hana Hoberg, Elizabeth Herron, LeeAnne Doyle, Rick Abraham, Mark Bentley, Po-ling Inglis, Maree Colosimo, Steven Lane, Patsy M. Yates

**Affiliations:** 1 Centre for Healthcare Transformation, School of Nursing, Queensland University of Technology, Brisbane, Australia; 2 Cancer and Palliative Care Outcomes Centre, Queensland University of Technology, Brisbane, Australia; 3 Cancer Care Services, Metro North Health, Herston, Queensland Australia; 4 School of Nursing, Queensland University of Technology, Brisbane, Australia; 5 Royal Brisbane and Women’s Hospital, Brisbane, Australia; 6 Faculty of Health, Human Performance Research Centre, INSIGHT Research Institute, University of Technology Sydney (UTS), Sydney, Australia; 7 Centre for Children’s Health Research, Children’s Health Queensland Hospital and Health Service, Queensland Children’s Hospital, 62 Graham St, South Brisbane, Brisbane, QLD 4101 Australia; 8 Child Health Research Centre, Faculty of Health, Medicine and Behavioural Sciences, University of Queensland, Brisbane, Australia; 9 St Vincent’s Private Hospital Northside, Brisbane, Australia; 10 ICON Cancer Care, Brisbane, Australia; 11 Mater Private, Brisbane, Australia; 12 QIMR Berghofer Medical Research Institute, Brisbane, Australia; 13 The University of Queensland, Brisbane, Queensland Australia

**Keywords:** Chemotherapy-related symptoms, Symptom burden, Longitudinal cohort study, Patient-reported outcomes, Quality of life

## Abstract

**Purpose:**

Individuals undergoing chemotherapy experience a range of symptoms that can significantly impact their quality of life and treatment outcomes; the prevalence and severity of which can change across their treatment journey. This study aimed to identify and describe the longitudinal symptom experience during the first four months of chemotherapy administration across a range of cancer diagnoses.

**Methods:**

A prospective longitudinal cohort study was conducted at a cancer centre in Queensland, Australia. Monthly surveys assessed symptoms, distress, and quality of life across 4 months of chemotherapy.

**Results:**

A total of 252 participants completed baseline surveys (mean age 61 ± 12.3 years; 65% female; 40% breast cancer). Participants reported a mean of 22.5 ± 9.0 symptoms at baseline, with a statistically significant reduction in symptoms throughout the study period 21.9 ± 9.2 symptoms (*p* = 0.002). Fatigue (93%), insomnia (80%), and pain (72%) were most common at baseline and remained highly prevalent throughout the time period. Severe fatigue (25%), pain (15%), and constipation (15%) reduced over time, while peripheral neuropathy and decreased sexual interest increased.

**Conclusion:**

This study has produced insights into the high number of symptoms experienced during the initial four months of chemotherapy. Given the impact of symptoms on treatment outcomes, there is a critical need for the development of appropriate assessment tools capable of capturing this broad range of patient symptom experiences.

**Implications for Cancer Survivors:**

Understanding symptom patterns over time can support early identification of high-risk patients, guide personalised supportive care strategies and optimise treatment tolerance and quality of life during and beyond chemotherapy.

**Supplementary Information:**

The online version contains supplementary material available at 10.1007/s00520-026-10955-w.

## Introduction

Over the past decade, cancer therapies have developed significantly, resulting in improved treatment outcomes and extended survival [1]. While these breakthroughs have given hope to many, patients undergoing chemotherapy continue to experience a high frequency of treatment-related symptoms, impacting their health status, quality of life (QoL), and outcomes [2–4]. The severity of these symptoms varies considerably between patients and throughout their treatment journey [5]. Symptoms frequently reported during chemotherapy include physical issues such as pain, fatigue, nausea, and vomiting, alongside psychological concerns such as depression and anxiety [6]. In addition, symptoms reported by people undergoing chemotherapy are often more severe and substantial than those experienced during other cancer treatments, such as radiation and hormone therapies [7]. Consequently, patients undergoing chemotherapy experience frequent and variable supportive care needs. Improving the management of the physical and psychosocial consequences of systemic anti-cancer therapies is consistently highlighted as an unmet need during survivorship [8]. These unmet needs are associated with reduced QoL, highlighting the need for comprehensive assessment and management of the symptom experience both during and following receipt of cancer therapies [8, 9].

While research on symptom experience and its impact on QoL and treatment outcomes is growing, historically, a high proportion of evidence comes from cross-sectional studies measuring symptoms at a single time point rather than longitudinal evidence [10]. The reliance on cross-sectional designs limits insight into symptom trajectories and overlooks the dynamic nature of treatment-related side effects and evolving supportive care needs [11]. Additionally, symptom experience in clinical trials is often reported by clinicians, who may underestimate the incidence and severity compared to patient-reported outcomes [12, 13]. While these gaps in both study design and reporting underscore the importance of patient-reported longitudinal assessment, current longitudinal data is limited in its ability to reflect the complexity and variability of symptom trajectories in routine clinical care.

Longitudinal evidence on symptom experience has predominantly focused on specific cancer populations (e.g. breast) and on identifying co-occurring symptoms such as depression, fatigue, sleep, and pain [14, 15]. Studies consistently show that during the first months of chemotherapy patients experience multiple concurrent symptoms, dominated by fatigue, gastrointestinal problems, pain, neuropathy, sleep disturbance, and psychological distress [16–18]. Patterns are broadly similar across cancer types, with lung cancers showing more respiratory symptoms, and gastrointestinal cancers more digestive burden [16, 19, 20].

This pattern is also reflected in haematological malignancies, with distinct symptom trajectories across the treatment continuum in lymphoma, with fatigue consistently emerging as the predominant symptom at each time point. Psychological symptoms including disturbed sleep and difficulty concentrating are also reported to substantially influence daily functioning [21]. These findings are derived from specific cancer types and timeframes and may not reflect the broader, real-world longitudinal data of the contemporary cancer care services. An additional challenge to symptom management lies in the reliance on symptom assessment tools that typically capture a limited number of predefined symptoms. Current commonly used tools typically assess 10–30 symptoms (e.g. The Memorial Symptom Assessment Scale, The Edmonton Symptom Assessment System, or MD Anderson Symptom Inventory), potentially underestimating or omitting significant treatment-related issues and contributing to unmet supportive care needs [21–23]. As a result, these instruments may fail to reflect the full range of experiences of patients undergoing chemotherapy or identify the symptoms patients feel are most burdensome. Therefore, more comprehensive approaches to symptom monitoring are beneficial to accurately reflect the diversity, co-occurrence, and variability of patient-reported symptoms over time.

The PRO-CTCAE (Patient-Reported Outcomes Common Terminology Criteria for Adverse) represents a comprehensive patient-reported tool that captures 78 symptom domains across 124 items [24]. It enables patients to self-report the frequency, severity, and interference with daily activities of treatment-related symptoms, and its use has been associated with improved symptom control [24]. Despite its utility, application of the PRO-CTCAE outside of clinical trial settings is limited, with existing research studies predominantly conducted in non-Western populations, restricted to single tumour streams, or on a limited subset of symptoms [25, 26]. As a result, robust longitudinal evidence describing comprehensive symptom profiles across diverse cancer types in real-world chemotherapy settings is lacking.

Understanding how patients’ symptoms and QoL evolve over time is critical to informing service delivery models and interventions that effectively address supportive care needs. Although robust symptom assessment has been shown to improve both QoL and survival, its consistent integration into routine oncology care remains limited [27]. This may be because tools developed for research in single, highly selected populations are not readily applicable or useful for guiding real-time clinical decision-making. Longitudinal evaluation of symptoms in real-world settings is therefore essential to understand changes throughout treatment and guide the development of interventions and services that respond to patients’ unmet needs [11]. This study aims to describe the longitudinal symptom experience of patients undergoing chemotherapy across diverse cancer types in a real-world Australian treatment setting, using the PRO-CTCAE to capture 78 patient-reported symptom domains over the first 4 months of chemotherapy.

## Methods

### Study design and participants

A prospective, longitudinal, observational cohort study design was used to identify and describe the changes in symptom experiences, levels of distress, and QoL during the first 4 months of chemotherapy administration. Participants were recruited between December 2017 and January 2020 and comprised adults undergoing chemotherapy at one cancer treatment unit in metropolitan Queensland, Australia. Participants were eligible for inclusion if they met the following criteria: aged 18 years or older; were initiating chemotherapy with curative intent at the hospital; and were competent to give informed consent. Participants were excluded if they had < 3 cycles of chemotherapy remaining, were being treated with palliative intent, had insufficient English proficiency to supply consent and understand the survey, or had impaired cognitive function. This study was performed in line with the principles of the Declaration of Helsinki. Ethical approval for this study was sought and obtained by the St Vincent’s Health Australia and Aged Care Committee Human Research Ethics Committee (Approval number 16/31, TARGET: individually tailored health care programme for cancer patients).

### Procedure

Participant eligibility was established prior to recruitment through screening the oncology admission records. Eligible participants received a letter inviting them to participate in the study, as well as a consent form and information materials. Patients were enrolled in the study if they completed the associated consent form and returned it to the research team.

Following enrolment, baseline questionnaires were intended to be completed as soon as possible after commencement of their first cycle of treatment and monthly electronic symptom questionnaires for 3 consecutive months thereafter. Up to two reminder prompts were sent via email and text message to complete the questionnaire. Follow-up phone calls were also made by the research team if a survey was not completed within three days of being administered. These calls checked whether the participant had received the email, and if the study team could assist with its completion. Three attempts at different times of the day were made to contact the participant.

### Data sources and variables

#### Demographic, clinical information, health, and lifestyle information

Sociodemographic variables were collected at baseline, including age, sex, marital status, education, income ($AUD), family/care responsibilities, ethnicity, living arrangements, and employment. A range of clinical characteristics including cancer type and time since diagnosis were also collected at baseline from patient medical records. Self-reported health and lifestyle information was collected at baseline including height (cm) and weight (kg), smoking status, usual alcohol consumption, engagement in exercise (frequency, intensity), and use of pain medications including both over-the-counter and prescription products.

Presence of comorbid conditions was measured using the Self-administered Comorbidity Questionnaire (SCQ) [28]. The SCQ contains 36 items about occurrence, treatment, and functional impact of 12 common comorbid conditions, including cancer [28].

The malnutrition screening tool (MST) was also used at baseline. The MST is a validated brief measure used to assess the risk of malnutrition [29]. The MST asks three questions including whether a person has lost weight without trying and if yes, how much, and whether they have been eating poorly due to decreased appetite [29].

#### Symptoms experience

Symptom experience was measured using the National Cancer Institute Patient-Reported Outcome Common Terminology Criteria for Adverse Event (PRO-CTCAE). The PRO-CTCAE is a validated tool developed for patient self-reporting of treatment-related side effects in cancer clinical trials [30]. This tool is based on the CTCAE which is commonly used by clinicians to document adverse effects in cancer treatment [24]. The PRO-CTCAE has demonstrated content validity [31], construct validity, reliability, responsiveness, equivalence, and acceptability across different modes of administration (web based, paper and interactive voice response systems) for a diverse range of cancer types [32–34].

The PRO-CTCAE item library comprises 124 items representing 78 symptomatic adverse event terms across oral, gastrointestinal, respiratory, cardio/circulatory, cutaneous, neurological, visual/perceptual, attention/memory, pain, sleep, mood, genitourinary, sexual, and miscellaneous. Each assessed across up to three attributes: frequency, severity, and interference with daily activities. Items were scored on five-point Likert scales (ranging from 0 to 4), with higher scores indicating greater symptom burden. For frequency participants ranked symptoms as never, rarely, occasionally, frequently, or constantly. For interference, participants ranked symptoms as not at all, a little bit, somewhat, quite a bit, or very much, and for severity, participants ranked symptoms as none, mild, moderate, severe, or very severe [24, 30, 35]. The PRO-CTCAE was administered at baseline and then monthly for three months thereafter via an online survey hosted by RedCap. This methodology was based on the results of a pilot study previously published by the research team [36], which determined that completion of an assessment tool on a weekly basis was too burdensome. Studies indicate that a monthly recall period is not inferior to daily reporting for data reliability [37]. PRO-CTCAE results were interpreted as per the previously published grading algorithm [38].

#### Quality of life

Quality of life was assessed using the assessment of quality of life (AQoL-4D) which measures four dimensions of QoL including independent living, relationships, mental health, and senses [39]. Participants self-reported 12 items (three items under each sub-scale) using a four-point scale (ranging from 1 to 4). The AQoL was developed using hospital in-patients with general medical conditions and community members and has demonstrated validity and reliability [40, 41]. Australian norms are available, derived from the Australian national survey of mental health and wellbeing [42].

#### Distress

Distress was measured at baseline and follow-up using the distress thermometer (DT), which is a brief screening tool that assesses distress levels participants have experienced in the past week. Participants scored a single item on an 11-point scale (ranging from no distress to extreme distress). The DT is sensitive to participants who do not meet anxiety or depression diagnoses [43] and has been validated for use with cancer patients [44].

### Data analysis

#### Variable transformation

Scores on the PRO-CTCAE were transformed using the composite grading algorithm for PRO-CTCAE established by Basch and colleagues [38], which has been assessed for validity, reliability, and sensitivity. The grading algorithm produces a single numerical grading score for adverse events, derived from multiple PRO-CTCAE items. The grading algorithm is scored from 0 to 3 representing none, mild, moderate, and severe symptom experience [38]. Scores on the grading algorithm were further collapsed to a binary variable (yes/no), representing those who did or did not experience adverse events. Participant height (cm) and weight (kg) information was used to generate a body mass index (BMI) score (kg/m^2^).

Regarding comorbidities, scores for responses on whether participants had any of the 12 comorbidities listed on the SCQ (excluding cancer), received treatment for them, and whether they limited daily activities were tallied to produce an overall SCQ score from 0 to 36 [28]. Individual scores on the MST questions were combined to create an overall MST score, which categorised participants as low, moderate, or high risk of malnutrition [29].

#### Presentation of data

Descriptive statistics were used to summarise data. Chi-square was used to explore group differences on categorical variables, or Fisher’s exact test if expected cell sizes were less than five. Group differences on continuous variables were explored using *t*-tests, or Mann-Whitney *U* test if the data were not normally distributed. Repeated measures ANOVA was also undertaken and the Huynh-Feldt correction factor was applied to account for the violation of sphericity, in which observations from the same participant are likely to be correlated [45].

PRO-CTCAE scores based on the composite grading algorithm were ranked at all time points according to the prevalence of the top 20 symptoms experienced. A ranking of one indicated the highest symptom prevalence. This enabled symptoms to be reported as percentage change in prevalence between time points 1 and 4. Similarly, the prevalence of Grade 3 (severe) symptom severity and the percentage change in the prevalence of Grade 3 symptom severity from time 1 to 4 were reported. Missing data were not included in analyses or descriptive summaries. All data analyses were undertaken using SPSS version 29 [46] and statistical significance was determined at a level of *p* < 0.05.

## Results

### Participants

Of the 312 eligible patients invited to participate between December 2017 and January 2020, 252 (81%) completed the baseline survey. Reasons for declining to participate included being too sick (*n* = 17), too overwhelmed (*n* = 7), no reason specified (*n* = 31), or other (*n* = 4). Most participants were female (*n* = 164, 65%), of British descent (*n* = 186, 76%), with a mean age of 61 ± 12 years. Participant demographics are presented in Table 1. Approximately 40% of participants had a diagnosis of breast cancer (*n* = 101), 10% had bowel cancer (*n* = 25), and 11% had lung cancer (*n* = 28). Most had been diagnosed within the past year (*n* = 187, 89%), with a mean age at diagnosis of 61 ± 12 years. More than half of participants had a family history of cancer (*n* = 142, 57%). More than three-quarters of participants were married or partnered (*n* = 192, 77%) and just under half were employed (*n* = 91, 36.7%). Almost half had an annual household income over $70,000 (AUD) (*n* = 118, 47%).

**Table 1:** Participant demographics and clinical characteristics (*n* = 252)

Demographic	*N* (%)
Sex	
Male	88 (34.9%)
Female	164 (65.1%)
Age (mean, SD)	61.0 ± 12.3
Cancer diagnosis	
Breast cancer	101 (40.2)
Bowel cancer	25 (10.0)
Lung cancer	28 (11.2)
Pancreatic cancer	19 (7.6)
Oesophageal cancer	18 (7.2)
Prostate cancer	9 (3.6)
Lymphoma	10 (4.0)
Ovarian cancer	7 (2.8)
Other	34 (13.5)
Age at cancer diagnosis (mean, SD)	60.1 ± 12.3
Time since cancer diagnosis—days (mean, SD)	274.0 ± 1219.2
Time since cancer diagnosis—years	
< 1 year	187 (89.0%)
1–2 years	14 (6.7%)
> 2 years	9 (4.3%)
Family history of cancer	
Yes	142 (57.0%)
No	97 (39.0%)
Unknown	10 (4.0%)
Marital status	
Never married/partnered	18 (7.2%)
Married/partnered	192 (76.8%)
Divorced/separated	24 (9.6%)
Widowed	16 (6.4%)
Living status	
Living with partner and/or others	216 (86.1%)
Live alone	33 (13.1%)
Ethnicity	
British/Scottish/Welsh/Irish	186 (75.9%)
European	30 (12.2%)
Indigenous Australian	1 (0.4%)
Asian	2 (0.8%)
Pacific Islander	1 (0.4%)
Other	25 (10.2%)
Do you have responsibilities for children at home	
No	194 (77.6%)
Yes	56 (22.4%)
Do you have responsibilities for elders at home	
No	244 (97.6%)
Yes	6 (2.4%)
Highest level of education	
None	1 (0.4%)
Primary	4 (1.6%)
Secondary	91 (36.4%)
Certificate/diploma	81 (32.4%)
Bachelor/post-graduate	73 (29.2%)
Employment	
Employed	91 (36.7%)
Self-employed	27 (10.9%)
Unemployed	10 (4.0%)
Retired	120 (48.4%)
Hours worked if employed (mean, SD)	33.1 ± 11.7
Hours worked if self-employed (mean, SD)	28.1 ± 15.1
Annual household income (AUD)	
< $30 K	32 (13.9%)
$30–70 K	80 (34.8%)
$70–100 K	37 (16.1%)
> 100 K	81 (35.2%)
Health characteristics (baseline)	
Height (cm) (mean, SD)	168.1 ± 9.1
Weight (kg) (mean, SD)	78.6 ± 20.3
Body Mass Index (BMI – kg/m^2^) (mean, SD)	27.7 ± 6.9
Smoking status	
Current	10 (4.1%)
Former	114 (46.7%)
Never smoked	120 (49.2%)
Alcohol consumption (drinks per week)	
None	140 (58.3%)
1–5	56 (22.3%)
6 or more	44 (18.3%)
Alcohol consumption (daily)	
None	67 (28.2%)
1–2	76 (31.9%)
More than 2	95 (39.9%)
Use of over-the-counter pain medications (measured at baseline)	
No	101 (40.2%)
Yes	150 (59.8%)
Use of strong pain medications (e.g. opioids)	
No	199 (79.6%)
Yes	51 (20.4%)
Use of complementary and alternative medicines (CAMs)	
No	69 (27.6%)
Yes	181 (72.4%)
Current level of exercise	
None	39 (15.8%)
Limited	128 (51.8%)
Regular	80 (32.4%)
Usual exercise intensity	
Low	112 (91.1%)
Moderate	10 (8.1%)
Vigorous	1 (0.8%)
Limited/low intensity exercise	43 (35.2%)
Regular/low intensity exercise	68 (55.7%)
Regular/moderate intensity exercise	9 (7.4%)
Usual exercise frequency (times/week)	
0	146 (57.9%)
1–3 times	31 (12.3%)
4 or more times	75 (29.8%)
Risk of malnutrition (MST)^a^	
Low	139 (66.2%)
Moderate	28 (13.3%)
High	43 (20.5%)
SCQ score^a^ at baseline (mean, SD)	5.1 ± 2.8
Presence of comorbidities^c^	
Yes	168 (67.2%)
No	70 (28.0%)

^a^The malnutrition screening tool (MST) generates a score to define whether a participant is at risk of malnutrition based on if they have lost weight, how much weight has been lost and whether they have experienced decreased appetite

^b^The Self-Administered Comorbidity Questionnaire (SCQ) score is a composite measure of 12 comorbidities experienced, if treatment is received and whether the problem causes a limitation to functioning. The maximum score possible is 36

^c^Consists of the comorbidities asked in the SCQ, but with the addition of a free text question to specify any additional comorbidities, and the exclusion of cancer, given all participants have a cancer diagnosis

### Clinical characteristics

The mean body mass index (BMI) of the sample was in the “overweight” category (27.7 ± 6.9). Just under half had never smoked (*n* = 120, 49%) and 58% (*n* = 140) did not consume alcohol on a weekly basis. Almost one-third of participants engaged in regular exercise (*n* = 80, 32%, > four times per week). Approximately one in three (*n* = 71, 24%) were at risk of malnutrition based on their MST score and three-quarters (*n* = 168, 67%) had additional comorbidities. Mean SCQ score was 5.1 (± 2.7) out of a possible score of 36 (with higher scores indicating increased comorbid burden).

### Symptom prevalence

The questionnaires assessed 54 different symptoms. At baseline, the average number of symptoms experienced by participants was 22.5 ± 9.0 and the most prevalent symptoms were fatigue (93%), insomnia (80%), pain (72%), sadness (70%), and problems tasting food or drink (68%).

Throughout the study, the number of symptoms experienced reduced to 21.9 ± 9.2 (*p* = 0.002). At the fourth time point, the most prevalent symptoms were fatigue (84%), pain (63%), insomnia (63%), problems tasting food or drink (59%), and aching muscles (59%).

#### Top three symptoms overall

Of the 54 symptoms assessed, fatigue, pain, and insomnia remained the top three prevalent symptoms present at both baseline and time point 4, affecting 84%, 63%, and 63% of participants, respectively, at the final time point. Of note, there was a significant reduction in the proportions of patients that experienced fatigue (93% to 84%, *p* = 0.005) and insomnia (80% to 63%, *p* =  < 0.001), although the proportion of people with these symptoms remained high. The prevalence of pain also reduced from 72% to 63%, but this was not statistically significant (*p* = 0.059) (see Table 2).

**Table 2:** Ranking of symptom experience and change in prevalence from Survey 1 to Survey 4. Asterix denote significant difference at *p < 0.05, **p < 0.01, and ***p < 0.001

Symptom	Survey 1 *n* = 252			Survey 4 *n* = 216			Change from Survey 1 to Survey 4					
	Rank	Prevalence (overall)	Prevalence (Grade 3)	Rank	Prevalence (overall)	Prevalence (Grade 3)	Relative change in prevalence (overall)	Absolute change in prevalence (overall)	*P* value^a^	Relative change in prevalence (Grade 3)	Absolute change in prevalence (Grade 3)	*P* value^c^
Fatigue	1	234 (92.9)	62 (24.6)	1	182 (84.3)	35 (16.2)	↓ 9.3%	↓ 8.6%	0.005**	↓ 34.2%	↓ 8.4%	.030*
Insomnia	2	202 (80.2)	35 (13.9)	3	135 (62.5)	21 (9.7)	↓ 22.1%	↓ 17.7%	<.001**	↓ 30.2%	↓ 4.2%	0.199
Pain	3	181 (71.8)	38 (15.1)	2	137 (63.4)	20 (9.3)	↓ 11.7%	↓ 8.4%	0.059	↓ 38.4%	↓ 5.8%	0.067
Sadness	4	177 (70.2)	17 (6.7)	7	117 (54.2)	10 (4.6)	↓22.8%	↓16.0%	<.001**	↓31.3%	↓2.1%	0.427
Problems tasting food or drink	5	172 (68.3)	33 (13.1)	4	128 (59.3)	21 (9.7)	↓13.2%	↓9.0%	0.053	↓26.0%	↓3.4%	0.310
Headache	6	166 (65.9)	3 (1.2)	18	97 (44.9)	2 (0.9)	↓31.9%	↓21.0%	< 0.001**	↓25.0%	↓0.3%	1.000
Constipation	7	165 (65.5)	37 (14.7)	17	99 (45.8)	11 (5.1)	↓30.1%	↓19.7%	<.001**	↓65.3%	↓9.6%	<.001**
Diarrhea	8	159 (63.1)	6 (2.4)	7	117 (54.2)	4 (1.9)	↓14.1%	↓8.9%	0.059	↓20.8%	↓0.5%	0.759
Anxiety	9	158 (62.7)	18 (7.1)	6	119 (55.1)	3 (1.4)	↓12.1%	↓7.6%	0.109	↓80.9%	↓5.7%	0.003**
Hair loss^b^	10	150 (59.5)	NA	13	107 (49.5)	NA	↓10.0%	↓16.8%	0.032*	NA	NA	NA
Decreased appetite	10	150 (59.5)	35 (13.9)	12	109 (50.5)	16 (7.4)	↓15.1%	↓9.0%	0.051	↓46.8%	↓6.5%	0.026*
Dry mouth	10	150 (59.5)	18 (7.1)	18	97 (44.9)	15 (6.9)	↓24.5%	↓14.6%	0.002**	↓2.8%	↓0.2%	1.000
Nausea	12	146 (57.9)	27 (10.7)	19	95 (44.0)	6 (2.8)	↓24.0%	↓13.9%	0.003**	↓73.8%	↓7.9%	<.001**
Bloating	12	146 (57.9)	15 (6.0)	11	111 (51.4)	10 (4.6)	↓11.2%	↓6.5%	0.163	↓23.3%	↓1.4%	0.545
Frequent urination	13	145 (57.5)	0	16	100 (46.3)	0	↓11.2%	↓19.5%	0.016*	NA	NA	NA
Abdominal pain	14	144 (57.1)	22 (8.7)	11	111 (51.4)	8 (3.7)	↓10.0%	↓5.7%	0.227	↓57.5%	↓5.0%	0.036*
Concentration	14	144 (57.1)	14 (5.6)	8	115 (53.2)	8 (3.7)	↓6.8%	↓3.9%	0.403	↓33.9%	↓1.9%	0.388
Aching muscles	15	140 (55.6)	18 (7.1)	5	127 (58.8)	9 (4.2)	↑5.8%	↑3.2%	0.513	↓40.1%	↓2.9%	0.232
Sudden urge to urinate	16	136 (54.0)	0	20	93 (43.1)	0	↓10.9%	↓20.2%	0.021*	NA	NA	NA
Dry skin	17	134 (53.2)	11 (4.4)	10	113 (52.3)	7 (3.2)	↓1.7%	↓0.9%	0.853	↓27.3%	↓1.2%	0.633
Memory	18	130 (51.6)	9 (3.6)	9	114 (52.8)	5 (2.3)	↑2.3%	↑1.2%	0.853	↓36.1%	↓1.3%	0.588
Heartburn	19	129 (51.2)	17 (6.7)	21	88 (40.7)	7 (3.2)	↓20.5%	↓10.5%	0.026*	↓52.2%	↓3.5%	0.096
Shortness of breath	20	125 (49.6)	16 (6.3)	14	105 (48.6)	11 (5.1)	↓2.0%	↓1.0%	0.853	↓19.1%	↓1.2%	0.692
Aching joints	21	118 (46.8)	13 (5.2)	15	103 (47.7)	9 (4.2)	↑1.9%	↑0.9%	0.853	↓19.2%	↓1.0%	0.667
Dizziness	22	103 (40.9)	13 (5.2)	25	75 (34.7)	5 (2.3)	↓15.2%	↓6.2%	0.182	↓55.8%	↓2.9%	0.148
Decreased sexual interest	23	96 (38.1)	22 (8.7)	31	59 (27.3)	21 (9.7)	↓28.4%	↓10.8%	0.014*	↑11.5%	↑1.0%	0.750
Neuropathy	23	96 (38.1)	10 (4.0)	4	128 (59.3)	22 (10.2)	↑55.6%	↑21.2%	< 0.001**	↑155.0%	↑6.2%	0.010*
Mouth or throat sores	24	95 (37.7)	11 (4.4)	34	52 (24.1)	5 (2.3)	↓36.1%	↓13.6%	0.002**	↓47.7%	↓2.1%	0.309
Blurry vision	25	92 (36.5)	5 (2.0)	22	86 (39.8)	5 (2.3)	↑17.8%	↑6.5%	0.504	↑25.0%	↑0.03%	1.000
Itchy skin	26	91 (36.1)	5 (2.0)	23	85 (39.4)	3 (1.4)	↑9.1%	↑3.3%	0.503	↓30.0%	↓0.6%	0.731
Cough	26	91 (36.1)	7 (2.8)	27	69 (31.9)	4 (1.9)	↓11.3%	↓4.2%	0.379	↓32.1%	↓0.9%	0.557
Depression	27	88 (34.9)	12 (4.8)	29	65 (30.1)	7 (3.2)	↓13.8%	↓4.8%	0.278	↓33.3%	↓1.6%	0.485
Difficulty swallowing	28	83 (32.9)	11 (4.4)	32	57 (26.4)	5 (2.3)	↓19.8%	↓6.5%	0.130	↓47.7%	↓2.1%	0.309
Unexpected or excessive sweating	29	82 (32.5)	8 (3.2)	28	68 (31.5)	8 (3.7)	↓3.1%	↓1.0%	0.843	↑15.6%	↑0.5%	0.803
Hot flashes	30	77 (30.6)	9 (3.6)	26	70 (32.4)	6 (2.8)	↑5.9%	↑1.8%	0.690	↓22.2%	↓0.8%	0.794
Watery eyes	31	75 (29.8)	7 (2.8)	24	80 (37.0)	7 (3.2)	↑24.2%	↑7.2%	0.115	↑14.3%	↑0.4%	0.792
Palpitations	32	74 (29.4)	5 (2.0)	36	48 (22.2)	4 (1.9)	↓24.5%	↓7.2%	0.091	↓5.0%	↓0.1%	1.000
Hoarse voice	33	68 (27.0)	5 (2.0)	34	52 (24.1)	4 (1.9)	↓10.7%	↓2.9%	0.524	↓5.0%	↓0.1%	1.000
Tinnitus	34	65 (25.8)	6 (2.4)	33	54 (25.0)	5 (2.3)	↓3.1%	↓0.8%	0.915	↓4.2%	↓0.1%	1.000
Shivering or shaking chills	35	62 (24.6)	6 (2.4)	39	44 (20.4)	5 (2.3)	↓17.1%	↓4.2%	0.319	↓4.2%	↓0.1%	1.000
Body odor	36	62 (24.6)	2 (0.8)	42	35 (16.2)	0	↓34.2%	↓8.4%	0.030*	↓100%	↓0.8%	0.502
Loss of control of bowel movements	37	61 (24.2)	0	40	43 (19.9)	0	↓17.8%	↓4.3%	0.316	NA	NA	NA
Hiccups	38	58 (23.0)	4 (1.6)	43	33 (15.3)	1 (0.5)	↓33.5%	↓7.7%	0.036*	↓68.8%	↓1.1%	0.380
Acne	38	58 (23.0)	1 (0.4)	37	46 (21.3)	5 (2.3)	↓7.4%	↓1.7%	0.738	↑475%	↑1.9%	0.100
Loss of control of urine	38	58 (23.0)	0	29	65 (30.1)	0	↑30.9%	↑7.1%	0.092	0%	0%	NA
Swelling	39	51 (20.2)	2 (0.8)	30	64 (29.6)	5 (2.3)	↑46.5%	↑9.4%	0.024*	↑187.5%	↑1.5%	0.257
Breast enlargement or tenderness	40	45 (17.9)	2 (0.8)	41	41 (19.0)	0	↑6.2%	↑1.1%	0.811	↓100%	↓0.8%	0.502
Cheilitis	41	44 (17.5)	5 (2.0)	38	45 (20.8)	1 (0.5)	↑18.9%	↑3.3%	0.408	↓75.0%	↓1.5%	0.224
Wheezing	42	41 (16.3)	1 (0.4)	47	25 (11.6)	1 (0.5)	↓28.8%	↓4.7%	0.183	↑25%	↑0.1%	1.000
Vomiting	43	39 (15.5)	2 (0.8)	46	28 (13.0)	2 (0.9)	↓16.1%	↓2.5%	0.508	↑12.5%	↑0.1%	1.000
Nosebleed	43	35 (13.9)	0	35	51 (23.6)	2 (0.9)	↑69.8%	↑9.7%	0.008**	↑200%	↑0.9%	0.212
Dysuria	44	29 (11.5)	5 (2.0)	44	30 (13.9)	3 (1.4)	↑20.9%	↑2.4%	0.486	↓30.0%	↓0.6%	0.731
Hand and foot syndrome	45	17 (6.7)	2 (0.8)	45	29 (13.4)	4 (1.9)	↑100%	↑6.7%	0.019*	↑137.5%	↑1.1%	0.421
Skin burns from radiation	46	7 (2.8)	1 (0.4)	48	15 (6.9)	1 (0.5)	↓62.3%	↓11.4%	0.047*	↑25%	↑0.1%	1.000

PRO-CTCAE scores based on the composite grading algorithm were ranked at Survey 1 and Survey 4 based on the prevalence of symptom experience. A ranking of one indicated the highest symptom prevalence. Given the mean number of symptoms experienced across all time points was 22.7 (± 9.3), we were particularly interested in the change of ranking and prevalence of the top 20 most frequently occurring symptoms and whether they remained, became or were no longer a top 20 symptom between Survey 1 and 4. Symptoms were also reported on with respect to the percentage change in prevalence of symptom experience, that is, the percentage change between percentage prevalence for symptom experience from Survey 1 to 4. Similarly, we also reported on the prevalence of Grade 3 symptom severity (i.e. severe) and the percentage change in prevalence of Grade 3 symptom severity from Survey 1 to 4

^a^Comparisons between Survey 1 and Survey 4 were made using χ^2^ tests, including Fisher’s exact test, where appropriate

^b^The greatest severity for hair loss is Grade 2

^c^*P* value for Grade 3 severity compared to all other categories

## Symptom severity

### Grade 3 symptoms

**Fig. 1:**
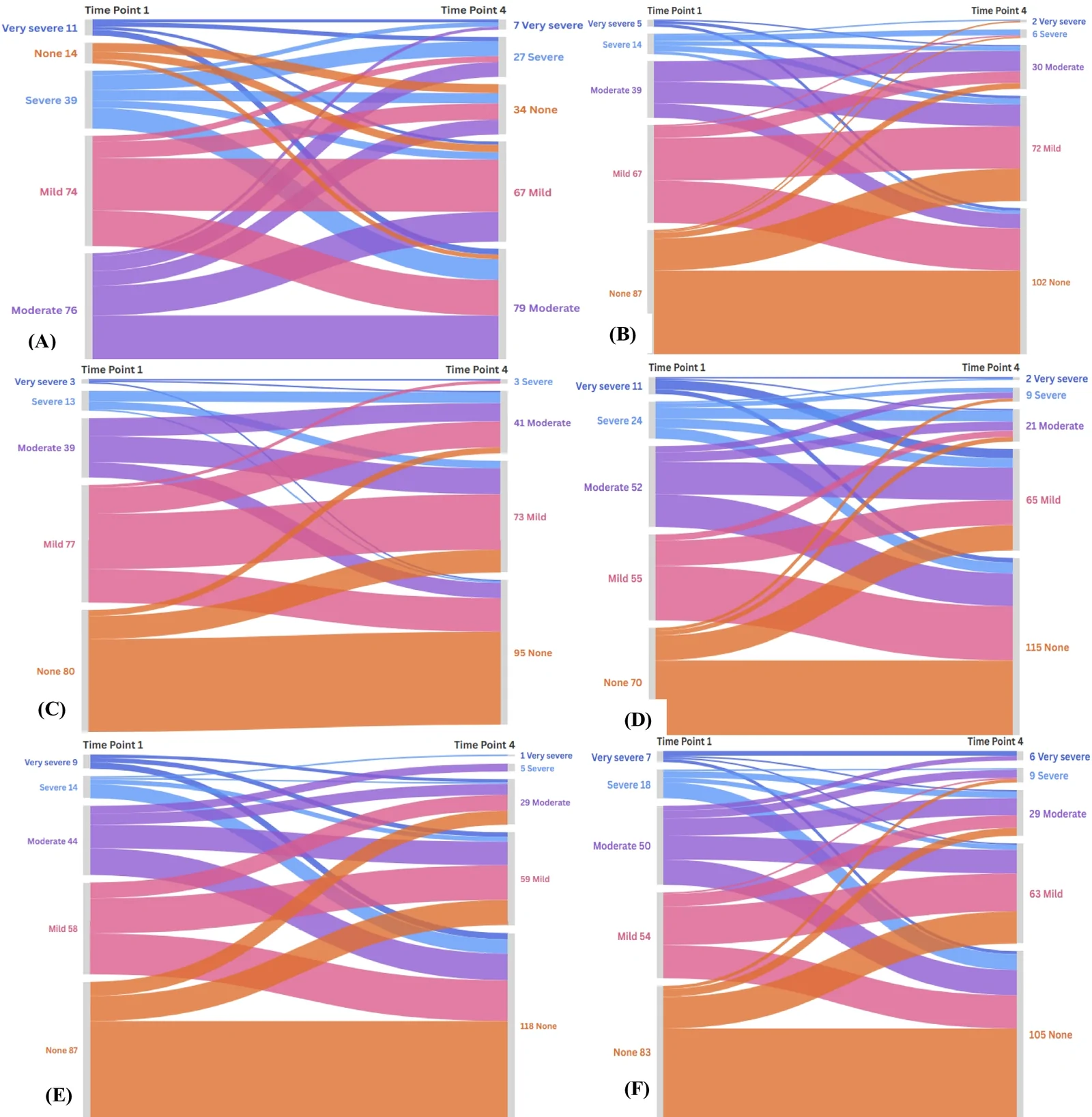
Depicts the symptoms that were ≥ Grade 3 and improved over time. **A** Fatigue (24.6% to 16.2%, *p* = 0.03), **B** abdominal pain (8.7 to 4.2%, *p* = 0.03), **C** anxiety (7.1 to 1.4%, *p* = 0.003), **D** constipation (14.7% to 5.1%, *p* =  < 0.001), **E** nausea (10.7 to 2.8%, *p* =  < 0.001), **F** decreased appetite (13.9 to 7.4%, *p* = 0.02)

**Fig. 2:**
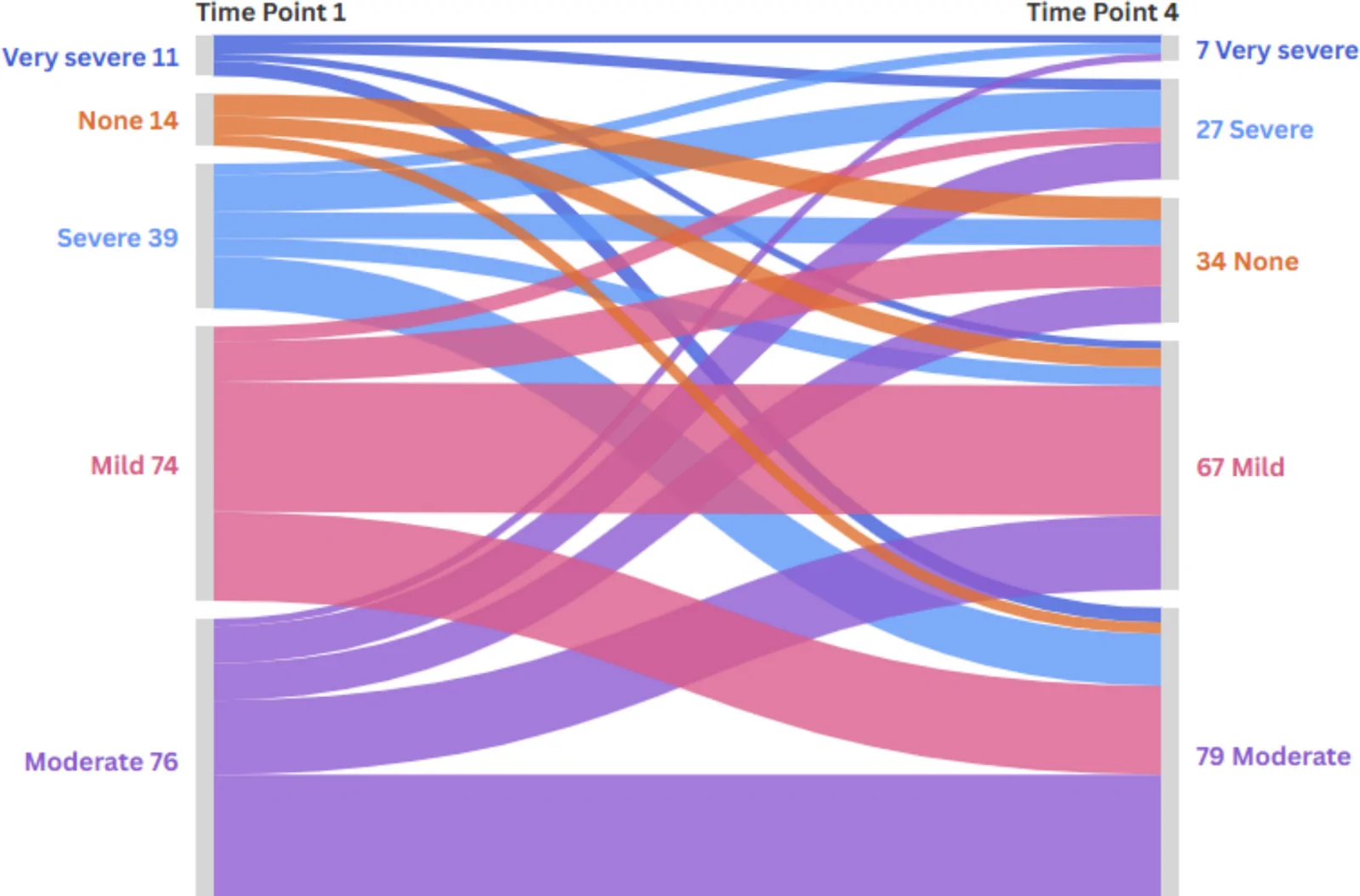
Depicts the symptoms that were ≥ Grade 3 and worsened over time. The prevalence of Grade 3 and above neuropathy increased over time (4.0% to 10.2% S4, *p* = 0.010)

While > 50% of patients experienced more than 20 symptoms at baseline, less than 25% experienced these at a severe (Grade 3) intensity or worse. The Grade 3 or worse intensity symptoms at baseline that affected more than 10% of patients included fatigue (25%), pain (15%), constipation (15%), insomnia (14%), decreased appetite (14%), problems tasting food and drink (14%), and nausea (11%) (see Table 2). There were significant improvements in Grade 3 intensity of fatigue, constipation, anxiety, nausea, decreased appetite, and abdominal pain; however, the proportion of patients experiencing Grade 3 intensity peripheral neuropathy increased significantly by the fourth time point (*p* = 0.010) (see Figs. 1 and 2).

### Quality of life and distress

Overall QoL improved significantly between time points one and four (*p* = 0.015), as well as the sub-scales of relationships (*p* < 0.001) and senses (*p* = 0.006). An improvement was also observed for mean distress scores which significantly improved from 3.6 ± 2.7 at baseline to 2.7 ± 2.7 at the last follow-up (*p* < 0.001). These data are presented in Table 3.

**Table 3:** Assessment of quality of life (AQoL) and symptom experience

	Survey 1 (*n* = 252) *M* (SD) *Mdn* (IQR)	Survey 2 (*n* = 233) *M* (SD) *Mdn* (IQR)	Survey 3 (*n* = 225) *M* (SD) *Mdn* (IQR)	Survey 4 (*n* = 216) *M* (SD) *Mdn* (IQR)	*P* value^a^	*P* value^b^
AQoL^c^ overall	.702 (±.214) .713 (.32)	.670 (±.235) .705 (.33)	.695 (±.219) .723 (.33)	.682 (±.226) .706 (.30)	.015*	.028*
AQoL IL subscale	.859 (±.182) .876 (.22)	.849 (±.197) .876 (.7)	.870 (±.177) .876 (.17)	.858 (±.191) .876 (.22)	.359	.344
AQoL REL subscale	.926 (±.110) .969 (.10)	.899 (±.132) .899 (.16)	.900 (±.126) .899 (.16)	.897 (±.130) .899 (.16)	<.001**	<.001**
AQoL SEN scale	.949 (±.069) 1.00 (.09)	.938 (±.079) .941 (.09)	.941 (±.068) .941 (.09)	.939 (±.071) .941 (.32)	.006**	.033*
AQoL MH scale	.887 (±.097) .893 (.10)	.881 (±.109) .893 (.12)	.891 (±.107) .912 (.13)	.887 (±.096) .892 (.10)	.564	.482
Distress thermometer score^d^	3.6 (± 2.7) 3.0 (5)	2.8 (± 2.5) 2.0 (5)	2.6 (± 2.4) 2.0 (4)	2.7 (± 2.7) 2.0 (4)	<.001**	<.001**
Number of symptoms experienced^e^	22.5 (± 9.0) 22.0 (13)	23.6 (± 9.2) 24.0 (13.8)	22.7 (± 9.6) 24.0 (14.0)	21.9 (± 9.2) 22.0 (13.0)	.223	0.002**

*AQoL*, assessment of quality of life; *IL*, independent living; *REL*, relationships; *SEN*, sensory; *MH*, mental health; *M*, mean; *Mdn*, median; *SD*, standard deviation; *IQR*, interquartile range

^a^Comparisons between Survey 1 and Survey 4 were conducted using paired samples test, with *p < 0.05, **p < 0.01, ***p < 0.001

^b^Comparisons across Surveys 1 to 4 were conducted using ANOVA repeated measures applying Huynh–Feldt correction factor, with *p < 0.05, **p < 0.01, ***p < 0.001

^c^The assessment of quality of life (AQoL) measure consists of an overall score with minimum and maximum possible scores of 12 and 48, respectively. Lower scores represent better quality of life than higher scores. The tool contains four subscales to measure the domains of independent living (IL), relationships (REL), sensory (SEN), and mental health (MH). Each subscale has a minimum and maximum possible score of zero and 12, respectively. Utility scoring has been applied as per http://www.aqol.com.au/index.php/norms

^d^The Distress Thermometer score measures how much distress is experienced over a 7-day period. The minimum and maximum possible scores are zero and ten, respectively

^e^The total number of symptoms experienced, regardless of level of severity or frequency and not including sex-specific variables. The maximum possible number of symptoms experienced is 54

## Discussion

This prospective, longitudinal cohort study demonstrates the need to understand the dynamic and changing symptoms experienced during treatment. The longitudinal data provides new insights into symptoms experienced during the first 4 months of chemotherapy, many of which are seldom assessed in current assessment tools [10, 47]. To our knowledge, this is the first study that reports on the PRO-CTCAE outcomes using the composite grading algorithm in a non-clinical trial setting. Findings revealed a substantial symptom burden in this sample, with a mean prevalence of 22.5 of the 54 screened symptoms. The most common symptoms during the first 4 months of chemotherapy were fatigue, insomnia, pain, sadness, and taste problems. Although the overall symptom burden decreased, the average number of symptoms remained high at 21.9 by the fourth time point. Given the high number of symptoms present in this study, current tools used clinically may underestimate or omit important symptoms, leading to incomplete clinical pictures, suboptimal patient care, and reduced patient outcomes [10, 47–49].

Findings demonstrated that overall QoL and distress scores significantly improved between time points one and four. This improvement in QoL and distress scores persisted despite the relatively high burden of symptoms experienced across the study period. The clinical relevance of the improvement in distress thermometer scores is unclear, as the mean scores at all time points were below the clinically actionable level of 4 [50]; however, there was a statistically significant reduction from time point one to time point four. Similarly, change in QoL did not surpass the clinically meaningful threshold of 0.06, despite statistical significance [51]. These improvements over time are supported by the wider literature which suggests that with effective supportive care interventions, it is possible for QoL and distress to improve despite the presence of symptoms [52, 53]. Further studies should explore the role of comprehensive supportive care and symptom management to address QoL and distress during chemotherapy administration.

Consistent with previous research, we identified the persistence of fatigue, pain, and insomnia as the three most frequently reported symptoms throughout the first 4 months of chemotherapy administration [54, 55]. Despite a statistically significant reduction in their prevalence over the time points, these symptoms remained highly prevalent and were among the four most common Grade 3 (severe) symptoms experienced, alongside constipation. These findings demonstrate the chronic nature of fatigue, pain, and insomnia during chemotherapy, as well as the tendency of these symptoms to manifest at severe levels and impacting QoL [56]. Importantly, literature suggests certain populations may be at higher risk of developing symptoms, such as elevated fatigue in postmenopausal women and those undergoing hormonal changes [57]. Pain has also been shown to be experienced at higher rates among those experiencing psychological distress [58]. This highlights the benefit of a comprehensive, patient-centred approach to symptom management, which integrates both physical and psychological symptoms and their impacts to patient QoL [48]. Furthermore, early assessment of these symptoms may enable early supportive care interventions [59].

Most symptoms reduced over the study; however, chemotherapy-induced peripheral neuropathy (CIPN) worsened, with a 55.6% relative increase in prevalence (21.2% absolute change) by the fourth time point. This is of importance due to the long-term health effects associated CIPN such as ongoing pain, sensory loss, reduced dexterity, and gait disturbances [60]. These effects predispose patients to higher risk of injury and falls as well as worsening QoL and daily functioning [60, 61]. There are currently limited treatment options for CIPN, suggesting a need for the early integration of supportive care strategies to optimise function and QoL [60].

Importantly, we identified a high symptom burden of non-specific symptoms such as aching muscles, reduced concentration, hiccups, and dry mouth. Literature indicates that vague, non-life-threatening symptoms are often deprioritised in clinical care despite significantly impacting QoL [62–65]. During treatment, clinicians often prioritise monitoring and managing potentially life-threatening side effects or those that could necessitate treatment discontinuation. Despite inadequate assessment of these symptoms, many can be improved with relatively simple lifestyle or supportive care strategies, such as regular exercise [65]. Collectively, this suggests a need for comprehensive assessment to identify and respond to non-specific symptoms.

There are several limitations to consider in interpreting the results of this study. Firstly, participants included in this study were all undergoing chemotherapy at one private hospital. While a diverse range of cancer diagnoses and treatments were included, some symptoms may have been a manifestation of their diagnosis not their treatment. Secondly, people attending a private cancer centre are typically of higher socio-economic status, suggesting that they may have greater access to supportive care services and social support [66]. Therefore, these results may not be reflective of the symptom experience of populations attending a public cancer service. As the purpose of this study was to represent the needs of a typical population of patients presenting for cancer treatment at a metropolitan hospital, the diversity of the sample included in this study enables a description of the types of needs and experiences that are common in a standard treatment setting on a day-to-day basis. Additionally, pre-chemotherapy treatment history, dose-density, cumulative exposure, and prior surgical intervention or radiation therapy, were not collected. It is acknowledged that these factors may influence symptom trajectories and the absence of this information limits the interpretation and generalisation of symptom profiles and trajectories in both directions. Residual effects of surgery or radiation may have elevated symptom burden at baseline and beyond, including symptoms such as lymphoedema, fatigue, skin reactions, peripheral numbness and tingling, dysphagia, and genitourinary or gastrointestinal disturbance. Conversely, participants with prior treatment experience may have developed effective supportive care strategies for managing symptoms such as nausea, pain, constipation, diarrhoea, insomnia, and psychological distress, potentially attenuating reported symptom burden relative to a treatment-naïve cohort. These influences cannot be determined from the available data, and both possibilities should be considered when interpreting the findings. Irrespective, this potential influence of prior treatment experience is reflective of the diverse needs and presentations of a real-world population that oncology health professionals may encounter in everyday practice. Lastly, it is possible that attrition in this study was not random. It is possible that patients with higher symptom burden may have been less likely to complete follow-up questionnaires. As missing data was treated by exclusion from analysis, the findings reflect only the subgroup with complete data than all participants originally enrolled. This may have resulted in an underestimation of symptom burden at later time points, and findings should be interpreted with this in mind.

The data reported in this study are reflective of only the first 4 months of receiving systemic cancer treatment. While this longitudinal study is different to existing evidence in terms of its real-world follow-up of patients over a 4-month period, the conclusions drawn are limited to this time period, and data collected. Continued symptom monitoring for the time following treatment completion may shed further light on the time course of symptom experiences and how best to care for patients following completion of first-line treatment. Further, we have not made any adjustment for the number of chemotherapy sessions a patient has had, nor the type of chemotherapy, as this may influence the type and severity of presenting symptoms (for example, the well-established relationship between peripheral neuropathy and platinum- or taxane-based chemotherapy [67]). Such factors are critical to explaining why some patients are more likely to experience higher levels of symptoms than others and provide further data that are needed to inform targeted interventions [67]. Furthermore, we focused on symptom prevalence; further research should determine whether the emergence or resolution of symptoms across the treatment period translates to clinically meaningful change. However, we note that the minimal meaningful individual-level change threshold for worsening is one point for all the composite scores measured in this research [68].

## Conclusion

This research has produced valuable longitudinal insights into the common and most burdensome symptoms experienced by patients attending a metropolitan cancer setting during the first 4 months of chemotherapy administration. Collectively these findings highlight the need for more robust assessment tools that are utilised early in patients’ treatment journey and used to inform referral management of treatment-related side effects. While there may be barriers to implementation of routine patient-reported measures into routine oncology care, the findings of this study provide evidence which supports the benefits of comprehensive assessment of patients to ensure optimal patient outcomes are achieved [69]. The findings of this study suggest that addressing a broader spectrum of patient-reported symptoms during cancer treatment, including those often overlooked, can provide important data to enhance quality of care and improve patient outcomes.

## Implications for practice

These findings underscore the importance of implementing robust assessment tools early in the patient care pathway to guide referral and management of treatment-related side effects. There is a clear need to expand symptom assessment beyond current assessment tools to enable tailored, comprehensive symptom management and optimise patient outcomes.

## Supplementary Information

Below is the link to the electronic supplementary material.

ESM 1 (DOCX 22.7 KB)

## Data Availability

No datasets were generated or analysed during the current study.
