# Supplementary material for: Symptoms experienced during the first 4 months of chemotherapy administration: a longitudinal cohort study in an Australian cancer treatment unit
Source: Support Care Cancer. 2026 Jul 3;34(7):729. doi: 10.1007/s00520-026-10955-w (PMC13331895; doi:10.1007/s00520-026-10955-w)
Supplement: Supplementary file 1 — (DOCX 22.7 KB) [file 520_2026_10955_MOESM1_ESM.docx]

**Supplementary Table 1: Assessment of Quality of Life (AQoL)**

| AQoL Question | Survey 1 | Survey 2 | Survey 3 | Survey 4 | p-value |  |  |
| --- | --- | --- | --- | --- | --- | --- | --- |
| Help looking after yourself (n, %) | | | | | | |  |
| 1 – no help | 204 (81.6%) | 183 (82.1%) | 177 (85.5%) | 163 (81.1%) | 0.36 |  |  |
| 2 – occasionally | 40 (16.0%) | 32 (14.4%) | 20 (9.7%) | 26 (13.1%) |  |  |  |
| 3 – need help | 5 (2.0%) | 4 (1.8%) | 8 (3.9%) | 9 (4.5%) |  |  |  |
| 4 – need daily help | 1 (0.4%) | 4 (1.8%) | 2 (1.0%) | 1 (0.5%) |  |  |  |
| Help when doing household tasks (n, %) | | | | | | |  |
| 1 – no help | 114 (45.4%) | 96 (43.4%) | 99 (47.8%) | 95 (47.5%) | 0.91 |  |  |
| 2 – occasionally | 82 (32.7%) | 67 (30.3%) | 65 (31.4%) | 67 (33.5%) |  |  |  |
| 3 – need help | 38 (15.1%) | 40 (18.1%) | 36 (17.4%) | 26 (13.0%) |  |  |  |
| 4 – need daily help | 17 (6.8%) | 18 (8.1%) | 7 (3.4%) | 12 (6.0%) |  |  |  |
| Ease getting around home and community (n, %) | | | | | | |  |
| 1 – can get around | 217 (86.8%) | 187 (84.2%) | 174 (84.5%) | 167 (83.5%) | 0.56 |  |  |
| 2 – difficult | 19 (7.6%) | 22 (9.9%) | 22 (10.7%) | 20 (10.0%) |  |  |  |
| 3 – cannot get around community/home difficult | 14 (5.6%) | 12 (5.4%) | 10 (4.9%) | 12 (6.0%) |  |  |  |
| 4 – cannot get around home or community | - | 1 (0.5%) | - | 1 (0.5%) |  |  |  |
| Because of health, relationships are generally (n, %) | | | | | | |  |
| 1 – very close and warm | 233 (92.8%) | 199 (89.6%) | 185 (88.9%) | 172 (86.0%) | **0.03*** |  |  |
| 2 – sometimes | 16 (6.4%) | 20 (9.0%) | 18 (8.7%) | 25 (12.5%) |  |  |  |
| 3 – seldom | 1 (0.4%) | 2 (0.9%) | 4 (1.9%) | 3 (1.5%) |  |  |  |
| 4 – no close and warm relationships | 1 (0.4%) | 1 (0.5%) | 1 (0.5%) | - |  |  |  |
| Relationships with other people (n, %) | | | | | | |  |
| 1 – plenty of friends, never lonely | 195 (78.6%) | 162 (73.3%) | 144 (69.6%) | 137 (68.5%) | 0.08 |  |  |
| 2 – have friends, occasionally lonely | 43 (17.3%) | 54 (24.4%) | 54 (26.1%) | 54 (27.0%) |  |  |  |
| 3 – some friends, often lonely | 8 (3.2%) | 3 (1.4%) | 8 (3.9%) | 7 (3.5%) |  |  |  |
| 4 – socially isolated and feel lonely | 2 (0.8%) | 2 (0.9%) | 1 (0.5%) | 2 (1.0%) |  |  |  |
| Health and relationship with family (n, %) | | | | | | |  |
| 1 – role in family unaffected by health | 149 (59.4%) | 106 (47.8%) | 103 (49.5%) | 95 (47.5%) | **0.04*** |  |  |
| 2 – parts of family role I can’t do | 88 (35.1%) | 94 (42.3) | 86 (41.4%) | 90 (45.0%) |  |  |  |
| 3 – many parts of family role I can’t do | 14 (5.6%) | 20 (9.0%) | 18 (8.7%) | 14 (7.0%) |  |  |  |
| 4 – can’t carry out family role | - | 2 (0.9%) | 1 (0.5%) | 1 (0.5%) |  |  |  |
| Vision (n, %) | | | | | | |  |
| 1 – I see normally | 162 (64.5%) | 119 (53.6%) | 105 (50.5%) | 106 (53.3%) | **0.02*** |  |  |
| 2 - I have some difficulty focusing on things | 88 (35.1%) | 101 (45.5%) | 99 (47.6%) | 90 (45.2%) |  |  |  |
| 3 – I have lots of difficulty seeing things | 1 (0.4%) | 2 (0.9%) | 4 (1.9%) | 3 (1.5%) |  |  |  |
| 4 – I only see general shapes, or am blind | - | - | - | - |  |  |  |
| Hearing (n, %) | | | | | |  |  |
| 1 – I hear normally | 191 (75.8%) | 172 (77.5%) | 161 (77.4%) | 152 (76.4%) | 0.99 |  |  |
| 2 – I have some difficulty hearing | 58 (23.0%) | 43 (19.4%) | 45 (21.6%) | 45 (22.6%) |  |  |  |
| 3 – I have difficulty hearing things clearly | 2 (0.8%) | 7 (3.2%) | 2 (1.0%) | 2 (1.0%) |  |  |  |
| 4 – I hear very little | 1 (0.4%) | - | - | - |  |  |  |
| Communicating with others (n, %) | | | | | | |  |
| 1 – I have no trouble speaking to them or understanding what they are saying | 234 (92.9%) | 197 (89.6%) | 190 (91.4%) | 177 (88.9%) | 0.15 |  |  |
| 2 – I have some difficulty being understood by people who do not know me | 15 (5.6%) | 22 (10.0%) | 18 (8.7%) | 22 (10.6%) |  |  |  |
| 3 – I am only understood by people who know me well | 3 (1.2%) | - | - | 1 (0.5%) |  |  |  |
| 4 – I cannot adequately communicate with others | - | 1 (0.5%) | - | - |  |  |  |
| Sleep (n, %) | | | | | | |  |
| 1 – I am able to sleep without difficultly most of the time | 66 (26.2%) | 59 (26.8%) | 62 (29.7%) | 67 (33.5%) | 0.29 |  |  |
| 2 – my sleep is interrupted some of the time | 84 (33.3%) | 67 (30.5%) | 66 (31.6%) | 62 (31.0%) |  |  |  |
| 3 – My sleep is interrupted most nights | 86 (34.1%) | 69 (31.4%) | 66 (31.6%) | 56 (28.0%) |  |  |  |
| 4 – I sleep in short bursts only | 16 (6.4%) | 25 (11.4%) | 15 (7.2%) | 15 (7.5%) |  |  |  |
| General feeling (n, %) | | | | | | |  |
| 1 – I do not feel anxious, worried or depressed | 109 (43.3%) | 95 (43.0%) | 99 (47.6%) | 87 (43.7%) | 0.56 |  |  |
| 2 – I am slightly anxious, worried or depressed | 116 (46.0%) | 102 (46.2%) | 89 (42.8%) | 90 (45.2%) |  |  |  |
| 3 – I feel moderated anxious, worried or depressed | 22 (8.7%) | 21 (9.5%) | 18 (8.7%) | 21 (10.6%) |  |  |  |
| 4 – I am extremely anxious, worried or depressed | 5 (2.0%) | 3 (1.4%) | 2 (1.0%) | 1 (0.5%) |  |  |  |
| Pain or discomfort (n, %) | | | | | | |  |
| 1 – no pain | 114 (45.6%) | 102 (46.2%) | 102 (48.8%) | 90 (45.2%) | 0.34 | | |
| 2 – moderate pain | 129 (51.6%) | 109 (49.3%) | 98 (46.9%) | 98 (49.3%) |  | | |
| 3 – severe pain | 7 (2.8%) | 10 (4.5%) | 8 (3.8%) | 11 (5.5%) |  | | |
| 4 – unbearable pain | - | - | 1 (0.5%) | - |  | | |

*Differences between Survey 1 and Survey 4 were made using χ^2^tests, including Fisher’s exact test, where appropriate*
